# Observation of sub-wavelength phase structure of matter wave with two-dimensional optical lattice by Kapitza-Dirac diffraction

**DOI:** 10.1038/s41598-020-62551-5

**Published:** 2020-04-03

**Authors:** Kai Wen, Zengming Meng, Pengjun Wang, Liangwei Wang, Liangchao Chen, Lianghui Huang, Lihong Zhou, Xiaoling Cui, Jing Zhang

**Affiliations:** 1 State Key Laboratory of Quantum Optics and Quantum Optics Devices, Institute of Opto-Electronics, Collaborative Innovation Center of Extreme Optics, Shanxi University, Taiyuan, 030006 P.R. China; 2 Beijing National Laboratory for Condensed Matter Physics, Institute of Physics, Chinese Academy of Sciences, Beijing, 100190 China; 3 Songshan Lake Materials Laboratory, Dongguan, Guangdong 523808 China

**Keywords:** Ultracold gases, Atom optics

## Abstract

We report an experimental demonstration of generation and measurement of sub-wavelength phase structure of a Bose-Einstein condensate (BEC) with two-dimensional optical lattice. This is implemented by applying a short lattice pulse on BEC in the Kapitza-Dirac (or Raman-Nath) regime, which, in the classical picture, corresponds to phase modulation imprinted on matter wave. When the phase modulation is larger than 2*π* in a lattice cell, the periodicity of phase naturally forms the sub-wavelength phase structure. By converting the phase information into amplitude, we are able to measure the sub-wavelength structure through the momentum distribution of BEC via the time-of-flight absorption image. Beyond the classical treatment, we further demonstrate the importance of quantum fluctuations in the formation of sub-wavelength phase structure by considering different lattice configurations. Our scheme provides a powerful tool for exploring the fine structure of a lattice cell as well as topological defects in matter wave.

## Introduction

The resolution of a conventional optical system is limited by the wavelength of light waves due to diffraction^1,2^. For a standing wave created by two laser fields with wavelength *λ*, the lattice spacing between adjacent intensity maxima is *λ*_*L*_/2, where *λ*_*L*_ = *λ*/*sin*(*θ*/2), with *θ* being the intersecting angle between the two fields. Therefore, the spatial resolution of interferometric lithography is always limited to *λ*_*L*_/2^3^. Circumventing this limit to create pattens with spatial resolution smaller than *λ*_*L*_/2 is not only interesting from a fundamental point of view, but is also relevant for the semiconductor industry. In the past decades, many schemes have been proposed to improve the spatial resolution of interferometric lithography beyond the diffraction limit, including the schemes of multiphoton nonlinear processes with classical light^4–6^, quantum lithography with quantum entanglement^7–10^, quantum dark state^11–13^, Rabi oscillations^14,15^, coherent atom lithography^16,17^.

**Figure 1:**
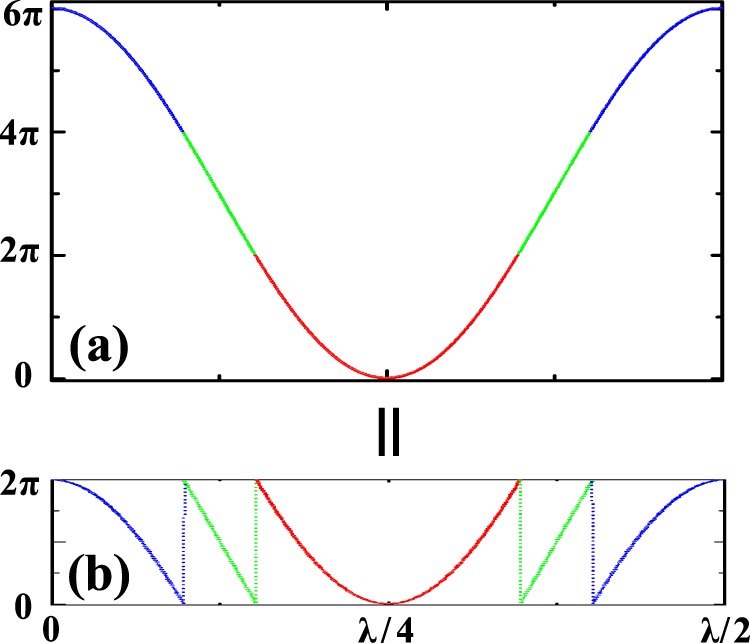
Schematic diagram of the sub-wavelength phase structure with in a lattice cell. (**a**) A continuous phase curve with amplitude larger than 2*π*. (**b**) The sub-wavelength phase structure with multiple 2*π* jumps.

In this paper, we experimentally develop and demonstrate a new scheme that generate sub-wavelength phase structure. In our work, a short pulse of optical lattice is illuminated on a Bose-Einstein condensate (BEC) working in the regime of Kapitza-Dirac (or Raman-Nath) scattering^18–22^. In this process, the lattice potential imprints a phase modulation on matter wave in position space. The picture is schematically shown in Fig. 1. For a continuous phase modulation larger than 2*π* within a lattice cell, as shown in Fig. 1(a), it can be equivalent to the phase curve as multiple 2*π* jumps forming a saw-tooth waveform in the principal value range of [0, 2*π*], as shown in Fig. 1(b). It manifests periodicity of phase as its natural feature. In this case, the phase curve forms the sub-wavelength phase structure, and the number *N* gives the sub-wavelength *λ*/(2*N*).

There are mainly two cases for the diffraction of matter-waves from a standing light wave^23^. The first case is the Bragg scattering, in which the atoms coherently transition between two resonantly coupled momentum state by the standing wave light^24–26^. In contrast, Kapitza-Dirac (or Raman-Nath) scattering can be diffracted into a number of momentum states, in which the interaction is sufficiently short and strong^18^. Originally predicted by Kapitza and Dirac for electrons^27^, it was first demonstrated with an atomic beam^18^, later with cold atoms^28^. With the realization of BEC, we can directly observe the dynamics of matter-wave diffraction in time-of-flight images^19–22^. Moreover, the diffraction of condensate atoms from standing light waves presents many applications in calibrating the lattices depth^19,21^, detecting the lattice structure^29^, performing high-resolution spectroscopy^30^ and metrology^31,32^.

Experimentally, the sub-wavelength phase structure is measured in momentum space via the time-of-flight absorption image, which corresponds to converting phase information into amplitude. We have observed different sub-wavelength pattens in the momentum distribution of BEC with two types of 2D optical lattices. The experimental findings can be well captured by the classical phase imprinting picture. Beyond this, the quantum fluctuation effect is also demonstrated in the formation of phase modulations with different lattice configurations.

## Results

### 2D optical lattice

**Figure 2:**
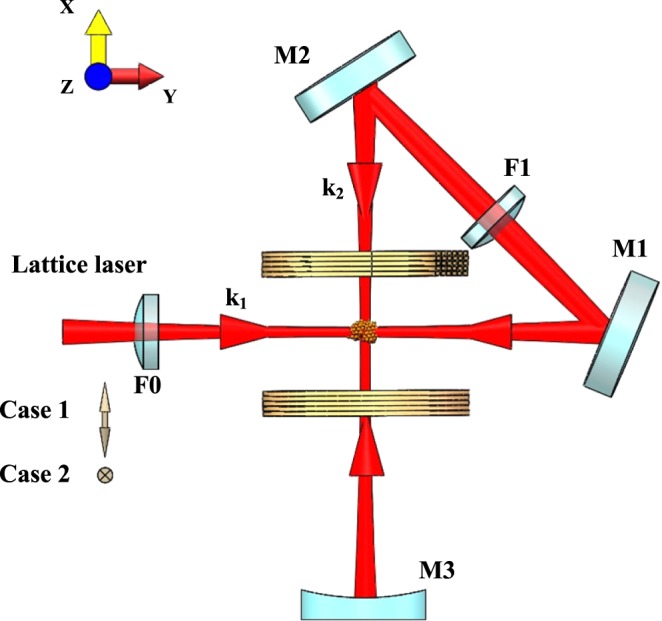
Schematic diagram of the experimental setup to realize the two-dimensional optical lattice. The two-dimensional optical lattices are made of a single fold retroreflected laser beam. The linear polarization of the incident laser beam aligned parallel (case 1) or normal (case 2) to the drawing plane can generate two different cases of 2D optical lattice potentials.

In order to generate two different cases of 2D optical lattice potentials, we employ the scheme of folded retroreflected mirrors, as shown in Fig. 2. This scheme has been used to experimentally design and implement the 2D optical lattice of double wells suitable for isolating and manipulating an array of individual pairs of atoms^33^ and predict a topological semimetal in the high orbital bands in this 2D lattice^34^. The light is folded by plane mirrors M1 and M2 and then retroreflected by concave mirror M3. The incoming beam with wave vector *k*_1_ is reflected by mirrors M1 and M2 and refocus on the atomic cloud with wave vector *k*_2_, and the two wave vectors intersect orthogonally. Lenses F0 and F1 with the concave mirror M3 generate almost the same focus beam radius at the intersection of the four beams with the 1/*e*^2^ radius of 200 *μ*m.

**Figure 3:**
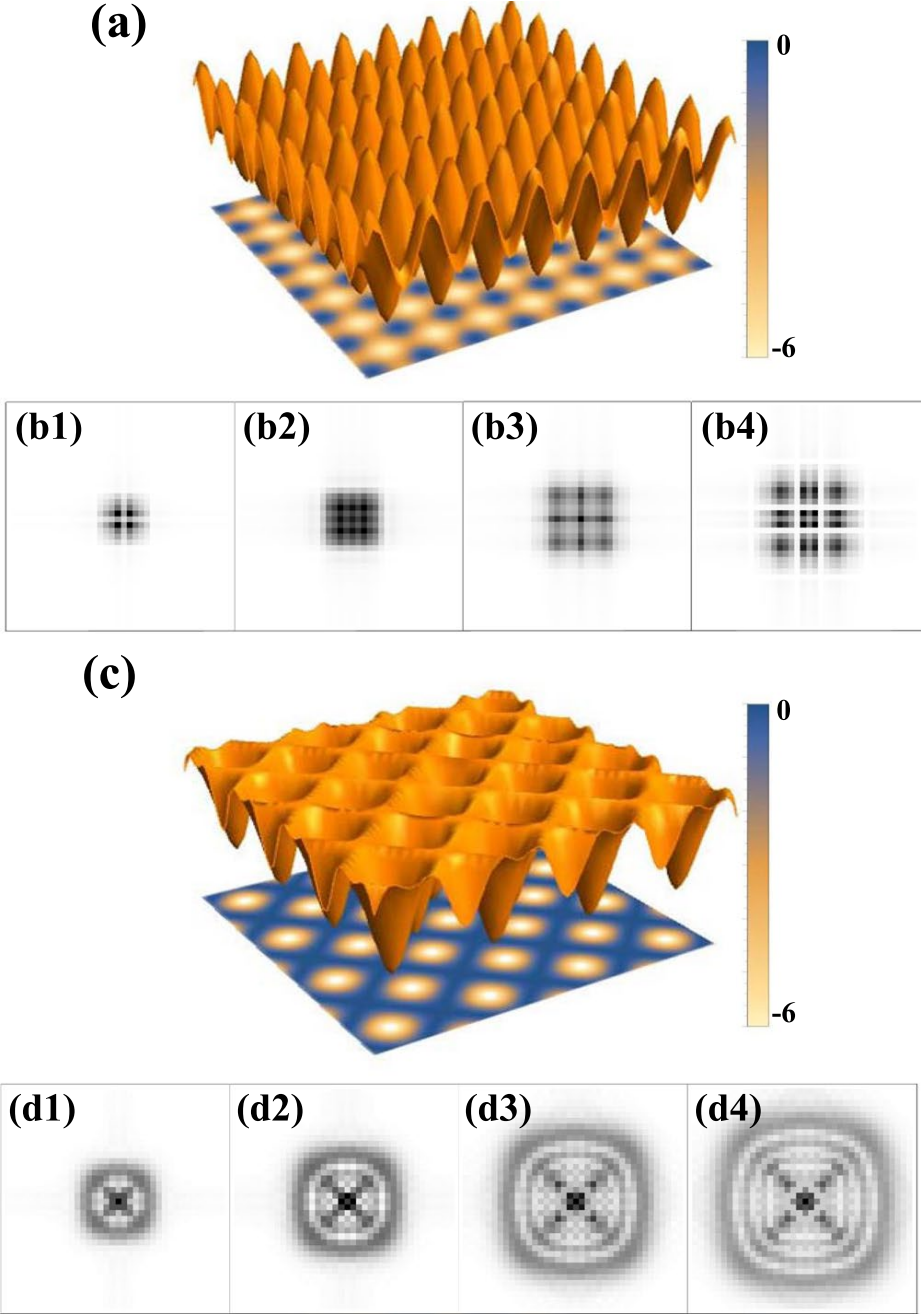
Two types of two-dimensional optical lattices and the associated momentum-space intensity distributions of BEC according to the phase modulation formula. (a) In-plane lattice (Eq. (1)). (**b1**–**b4**) Momentum-space intensity distribution of BEC with in-plane lattice pulse for *Vτ*/*ℏ* = 4, 6, 8, 10. (**c**) Out-plane lattice with red detuning (Eq. (2)). (**d1**–**d4**) Momentum-space intensity distribution of BEC with out-plane lattice pulse for ∣*V*∣*τ*/*ℏ* = 4, 6, 8, 10.

We first consider the case 1 when the linear polarization of incident laser beam is aligned parallel to the drawing plane (referred as in-plane lattice). Due to the orthogonal intersection of laser beam and the orthogonality of the polarization between *k*_1_ and *k*_2_, the resulting 2D lattice is a square lattice formed by two independent 1D lattices. The potential of 2D square lattice is described by

1 $${U}_{1}(x,y)=V[{\cos }^{2}(kx)+{\cos }^{2}(ky)].$$

here *k*_*r*_ = 2*π*/*λ* and *λ* denotes the wavelength of the lattice beam. This generates a 2D square lattice with antinode (nodes) spaced by *λ*/2 along x and y directions respectively, as shown in Fig. 3(a).

In the case 2, the linear polarization of incident laser beam is aligned normal to the drawing plane (referred as out-plane lattice). In this case the potential is not simply a sum of independent lattices in x and y, but is rather given by

2 $${U}_{2}(x,y)=V{[\cos (kx)+\cos (ky)]}^{2}.$$

The extra interference term, $2\,\cos (kx)\cos (ky)$ in Eq. (2), induces the lattice period of $\lambda /\sqrt{2}$ along *x* + *y* and *x* − *y* directions. When the lattice laser is red detuned (*V* < 0), the generated lattice is just like to dug the holes on the ground and there are nodal lines along the diagonals as shown in Fig. 3(c). Different from the square lattice in Eq. (1), the out-plane lattice in Eq. (2) displays different shapes near the antinode and node. We will show later that this property would significantly affect the sub-wavelength phase structure as measured in our experiment.

### Detection of sub-wavelength phase structure

When working in the Kapitza-Dirac regime with a short pulse duration *τ* ≪ *h*/4*E*_*rec*_, where *E*_*rec*_ is the photon recoil energy, the kinetic energy can be neglected and the lattice potential simply acts as a phase grating on the matter wave^20^. In this case, the evolution of BEC can be described classically by

3 $$\Psi (x,y)={\psi }_{0}{e}^{iU(x,y)\tau /\hslash }.$$

Here *ψ*_0_ is the Gaussian wave function of initial state of BEC, while the lattice potential *U*(*x*, *y*) imprints a phase modulation on the BEC. Take the in-plane lattice for example, when ∣*V*∣*τ*/*ℏ* > 2*π* the phase of BEC within a unit cell appears as multiple 2*π* jumps. Similarly, the phase structure occurs for out-plane lattice when ∣*V*∣*τ*/*ℏ* > *π*. Such 2*π* multiple phase jumps equivalently generate the sub-wavelength phase structure, as schematically shown in Fig. 1, where the number *N* gives the sub-wavelength *λ*/(2*N*). In order to deeply understand the sub-wavelength phase structure and sub-wavelength periodic structure, the atomic wavefunction after the lattice pulse with time interval *τ* can be transformed into discrete momentum, for example for the in-plane lattice,

4 $$\begin{array}{lll}\Psi (x,y) & = & {\psi }_{0}{e}^{i\frac{V\tau }{\hslash }{\cos }^{2}(ky)}{e}^{i\frac{V\tau }{\hslash }{\cos }^{2}(kx)}\\  & = & {\psi }_{0}{e}^{i\frac{V\tau }{\hslash }{\cos }^{2}(ky)}{e}^{i\frac{V\tau }{2\hslash }}{e}^{i\frac{V\tau }{2\hslash }\cos (2kx)}\\  & = & {\psi }_{0}{e}^{i\frac{V\tau }{\hslash }{\cos }^{2}(ky)}{e}^{i\frac{V\tau }{2\hslash }}{\sum }_{n=-\infty }^{+\infty }{(i)}^{n}{J}_{n}\left(\frac{V\tau }{2\hslash }\right){e}^{2nkx}.\end{array}$$

where *J*_*n*_ is Bessel function. Here, x and y directions are separable for in-plane lattice, which make ease to calculate. Thus the atomic wavefunction after the lattice pulse breaks up into the discrete momentum components of 2*nℏk*_*r*_ (n is integer) with population probability given by

5 $${P}_{n}={J}_{n}^{2}\left(\frac{V\tau }{2\hslash }\right).$$

when the interval time *τ* equals 2 × 2.4048*ℏ*/*V*, *J*_0_(2.4048) = 0 and the order *n* = 0 momentum component first vanishes. The lattice depth *V* = 2 × 2.4048*ℏ*/*τ* can be calibrated precisely by measuring the interval time *τ* at which the order *n* = 0 first vanishes. In this work, we mainly consider the case of ∣*V*∣*τ*/*ℏ* > 2*π*. It is natural that phase presents periodicity when phase is larger than 2*π*. Therefore, the atomic wavefunction after the lattice pulse should display this periodicity, as we know Bessel function has periodicity.

Since we can not directly measure the phase structure of BEC by the in-situ imaging, we must convert the phase information into amplitude. In other words, we try to measure this periodicity based on the discrete momentum components such as Eq. (4). The easiest way to realize this goal is to take the Fourier transform

6 $$\Psi ({p}_{x},{p}_{y})=F[\Psi (x,y)].$$

This transformation converts the phase information of real-space wave function to the amplitude information in momentum space. The resulted intensity distribution ${I}_{{p}_{x},{p}_{y}}=| \Psi ({p}_{x},{p}_{y}){| }^{2}$ in momentum space can be directly measured in the ultracold atomic experiment through the time-of-flight absorption imaging.

In Fig. 3(b1–b4),(d1–d4), we show the intensity distributions ${I}_{{p}_{x},{p}_{y}}$ from Eqs. (3) and (6) corresponding to the two different lattice potentials as displayed, respectively, in Fig. 3(a,c). Starting with the initial BEC (see details in the Methods section) which has a single momentum component (*k* = 0), the phase modulations with the 2D optical lattices generate discrete momentum components. Remarkably, these discrete components organize themselves into different patterns in a larger momentum scale, which is related to sub-wavelength phase structure in a smaller length scale (within a single cell). Since the in-plane lattice generates the sub-wavelength phase structure with the trenches along x and y (Fig. 3(a)), the intensity distribution after the Fourier transform presents the larger line structures along x and y, see Fig. 3(b1–b4). In contrast, out-plane lattice generates the sub-wavelength phase structure with ring structure (Fig. 3(c)) and the intensity distribution after the Fourier transform presents the larger ring structures, see Fig. 3(d1–d4).

**Figure 4:**
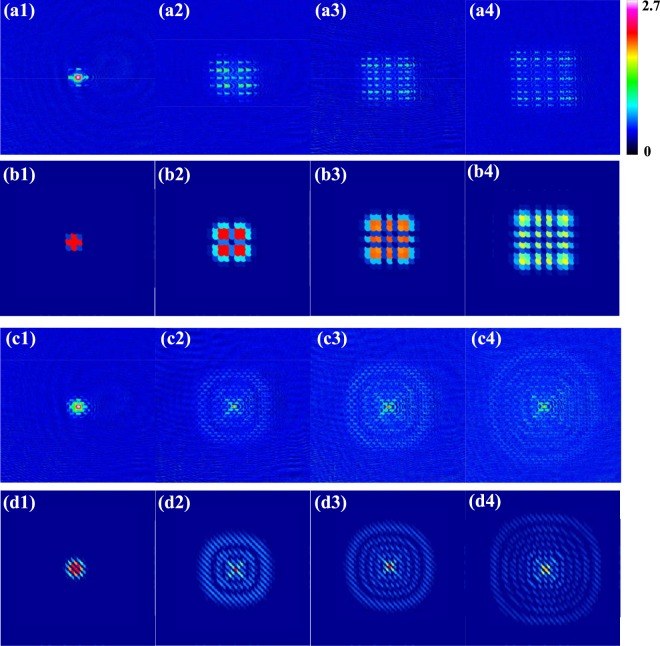
Atomic density distribution of the time-of-flight absorption image after applying 2D optical lattice pulse on BEC for red detuning (*V* < 0). (**a1–a4**) Experimental data for in-plane lattice pulse with ∣*V*∣/*E*_*rec*_ =  10, 59, 88, 123, in comparison to the theoretical results from full quantum evolutions (**b1–b4**). (**c1–c4**) Experimental data for out-plane lattice pulse with ∣*V*∣/*E*_*rec*_ =  10, 59, 88, 123, in comparison to the theoretical results from full quantum evolutions (**d1–d4**) . The lattice wavelength is *λ* = 800 nm. The duration time of applying 2D optical lattice pulse on BEC is 4 *μs* and TOF=7 ms.

In the experiment, *h*/4*E*_*rec*_ = 70 *μs* for the lattice wavelength of *λ* = 800 nm, which is red detuning corresponding to *V* < 0 in Eqs. (1) and (2). We apply 2D optical lattice short pulse for 4 *μs* on BEC, which work in the Kapitza-Dirac regime. Then we immediately turn off the optical trap, let the atoms ballistically expand in 7 ms and take the absorption image. Figure 4(a1–a4) show the atomic density distribution of the time-of-flight absorption image after applying in-plane lattice pulse on BEC and Fig. 4(c1–c4) are for out-plane lattice. These experimental data are in good consistence with theoretical results from the full quantum evolution of the BEC (see appendix), as shown by Fig. 4(b1–b4,d1–d4). The results are also qualitatively agreement with those from the classical treatment (Eq. (3)) (see appendix).

As expected, the atomic density distributions of the time-of-flight absorption image exhibit discrete momentum components. Nevertheless, the distribution of the discrete momentum components is not uniform, which depends on the sub-wavelength phase structure in a lattice cell. On a larger momentum scale, the distribution of discrete momentum components shows line structure along x and y for in-plane lattice, and ring structure for out-plane lattice. As increasing the lattice laser power, more and more lines or rings appear, see Fig. 4. This can be well explained by the phase picture since more and more 2*π* phase jumps occurs within the single cell by increasing ∣*V*∣, giving more delicate structure with smaller sub-wavelength phase structure. In this way, the line or ring patterns in a large momentum scale of the intensity distribution directly reflect the sub-wavelength phase structure of particular lattice potentials.

**Figure 5:**
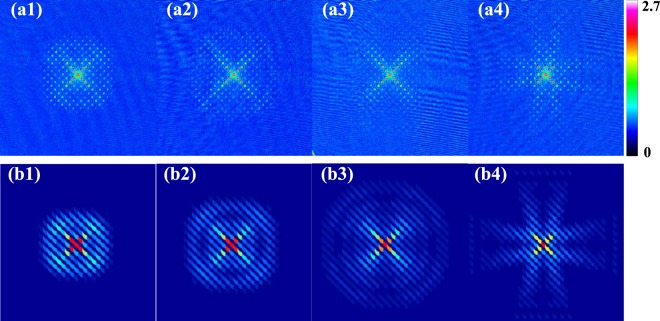
Atomic density distribution in the time-of-flight absorption image after applying 2D out-plane lattice with blue detuning. (**a1–a4**) Experimental data with *V*/*E*_*rec*_ = 34, 53, 82, 120, in comparison to the theoretical results from full quantum evolutions (**b1–b4**). The lattice wavelength is *λ* = 793.4 nm. The duration time of applying 2D optical lattice pulse on BEC is 4  *μs* and TOF = 7 ms.

To highlight the quantum fluctuation effect in affecting the phase formation, we have performed an additional set of experiments with the out-of-plane lattice pulse switching to blue detuning, i.e., with *V* > 0 in Eq. (2). The resulted momentum distribution obtained from the time-of-flight measurement is shown in Fig. 5. Contrary to the red detuning (*V* < 0) case (Fig. 4), here the ring structure gradually disappears as increasing ∣*V*∣, see Fig. 5(a1–a4). This phenomenon violates the prediction from classical treatment (Eq. (3)), and we attribute it to the enhanced quantum fluctuation effect under such lattice potential. Namely, the bottom of such lattice is fairly smooth and the quantum motion of the wave-packet due to kinetic term cannot be neglected, thereby driving the system outside the Kapitza-Dirac regime and invalidating the classical phase imprinting picture. In comparison, lattices *U*_1_ and *U*_2_ (Fig. 3(a,c)) exhibit sharp structures near the bottom, thus the low-energy states are well localized and able to reflect the structure of lattice potentials. In this case, the quantum motion of matter-wave is well suppressed and the classical treatment is qualitatively correct. More detailed comparison of BEC momentum distributions under various lattice configurations can be found in the appendix.

## Discussion

We have experimentally realized the sub-wavelength phase structure in BEC with 2D optical lattices. This is implemented by applying a short lattice pulse in the Kapitza-Dirac regime, such that in the classical picture the lattice potential imprints a phase modulation on matter wave. We have detected the sub-wavelength phase structure for two types of lattice potentials by measuring the intensity distribution of BEC in momentum space, which exhibits the line or ring patterns in a larger momentum scale. Our scheme can be used to detect more delicate properties of the optical lattice, such as the fine structure within a unit cell and its associated flux^35–37^. Finally, the sub-wavelength phase structure in our work can also be connected to the topological defects in matter wave^38–40^, such as phase steps or vortices, which could be equally detected in the momentum space.

Furthermore, we work beyond the classical regime and demonstrate the destructive effect of quantum fluctuations in the formation of sub-wavelength structure with a different lattice configuration. The quantum fluctuation generated by the kinetic term will play essential roles for the delocalized low-energy states, as generated by the blue-detuned optical lattices. As the atoms are mostly populated in the low-energy states but less in high-energy ones, the classical treatment breaks down and the large phase modulation can no longer be achieved.

## Methods

In our experiment, the ultracold ^87^Rb atoms in the $\left|F=2,{m}_{F}=2\right\rangle$ state is prepared in the crossed optical dipole trap^41,42^. Here *F* denotes the total angular momentum and *m*_*F*_ the magnetic quantum number of the state. Forced evaporation in the optical trap is used to create the BEC with up to 5 × 10^5^ atoms, which is used as the coherent matter wave. The lattice beam is derived from a single frequency Ti:sapphire laser and operated at a wavelength of *λ* = 800 nm. An acousto-optical modulator is used to control the intensity of the lattice beam. Then the light is coupled into a polarization maintaining fiber to provide a clean *TEM*_00_ spatial mode. A polarizer after the fiber creates a well defined polarization in the x-z plane. The two perpendicular lattices formed from a single folded retroreflected beam have intrinsic topological phase stability, which do not need the active phase locking. The coherence or the lifetime of atoms in lattices can reach more than 100 ms.

## Supplementary information

Supplementary Information.

## Data Availability

All data generated or analysed during this study are included in this published article. Additional data are also available from the corresponding authors upon reasonable request.
