# Supplementary material for: Observation of sub-wavelength phase structure of matter wave with two-dimensional optical lattice by Kapitza-Dirac diffraction
Source: Sci Rep. 2020 Apr 3;10:5870. doi: 10.1038/s41598-020-62551-5 (PMC7125164; doi:10.1038/s41598-020-62551-5)
Supplement: Supplementary file 1 — Supplementary Information. [file 41598_2020_62551_MOESM1_ESM.pdf]

SUPPLEMENTARY MATERIALS

Observation of sub-wavelength phase structure of matter wave with two-dimensional optical lattice by Kapitza-Dirac diffraction

Kai Wen,[1] Zengming Meng,[1] Pengjun Wang,[1] Liangwei Wang,[1] Liangchao Chen,[1] Lianghui Huang,[1] Lihong Zhou,[2] Xiaoling Cui,[2,3] and Jing Zhang[1]

[1]*State Key Laboratory of Quantum Optics and Quantum Optics Devices, Institute of Opto-Electronics, Collaborative Innovation Center of Extreme Optics, Shanxi University, Taiyuan 030006, P.R.China*
[2]*Beijing National Laboratory for Condensed Matter Physics, Institute of Physics, Chinese Academy of Sciences, Beijing 100190, China*
[3]*Songshan Lake Materials Laboratory , Dongguan, Guangdong 523808, China*

In this supplementary material, we provide details on the exact solution of BEC density distribution after a short lattice pulse, and compare exact results to those from classical treatment (Eqs. 3, 6 in main text) for various lattice configurations.

EXACT SOLUTION OF MOMENTUM DISTRIBUTION

The atoms in a two-dimensional (2D) optical lattice can be described by the Hamiltonian:

$$H_i = \frac{\mathbf{p}^2}{2m} + V[\cos^2(k_r x) + \cos^2(k_r y)], \tag{1}$$

$$H_o = \frac{\mathbf{p}^2}{2m} + V[\cos(k_r x) + \cos(k_r y)]^2. \tag{2}$$

Here H_i and H_o are, respectively, the Hamiltonian for in-plane and out-plane lattices. $k_r = 2\pi/\lambda$ is the recoil momentum, which defines the recoil energy as $E_{rec} = \hbar^2 k_r^2/(2m)$.

According to the Bloch theorem, the eigen-state wave function can be expressed as:

$$|n\mathbf{k}>= \frac{1}{\sqrt{S}} \sum_{\mathbf{G}} a_{n\mathbf{k}}(G_x, G_y) e^{i(k_x+G_x)x + i(k_y+G_y)y} \tag{3}$$

here $\mathbf{k}$ lies within the first Brillouin zone (BZ) and n is the band index; $G_{x,y}$ are the reciprocal vectors: $G_{x,y} = 2Nk_r$ for in-plane lattice and $G_{x,y} = N_{x,y}k_r$ for out-plane lattice (N, N_x, N_y are all integers).

Given the initial state as a Bose condensate at $|\mathbf{p}_0 = (0,0)>$, at time t the atomic density distribution in momentum space can be derived as:

$$\begin{aligned} n(p_x, p_y, t) &= |\sum_{n\mathbf{k}} e^{-iE_{n\mathbf{k}}t/\hbar} < \mathbf{p}|n\mathbf{k}>< n\mathbf{k}|\mathbf{p}_0 = (0,0)> |^2 \\ &= |\sum_{n}\sum_{\mathbf{G}} e^{-iE_{n0}t/\hbar} \delta_{p_x,G_x}\delta_{p_y,G_y} a_{n0}(G_x,G_y) a^*_{n0}(0,0)|^2 \end{aligned} \tag{4}$$

here $E_{n\mathbf{k}}$ is the eigen-energy of state $|n\mathbf{k}>$.

In our practical numerical calculations, we have set a cutoff momentum to both G_x and G_y as $20k_r$. Accordingly, the single-particle Hamiltonian is expanded as a $(2N_c+1)^2 \times (2N_c+1)^2$ matrix with $N_c = 20$. We have checked that such cutoff can give convergent results to the momentum distribution.

COMPARISON BETWEEN DIFFERENT LATTICE CONFIGURATIONS: EXACT SOLUTION VS. CLASSICAL TREATMENT

The density distribution in momentum space by the classical treatment (as shown in Eqs. 3, 6 in main text):

$$I_{p_x,p_y}(t) = |F[Ae^{iU(x,y)t/\hbar}]|^2 \tag{5}$$

here F means taking the Fourier transform, and $U(x,y)$ is the 2D lattice potential.

In Figs. 1-3, we show the momentum distributions from exact solutions and from classical treatments for various lattice pulse configurations, using the same parameters as in the experiments (see Fig. 4 and Fig. 5 in the main text).

1. In-plane lattice:

In Fig. 1 we compare the density distributions for in-plane lattice, respectively by the classical treatment [(a1)-(a4)] and exact solution [(b1)-(b4)].

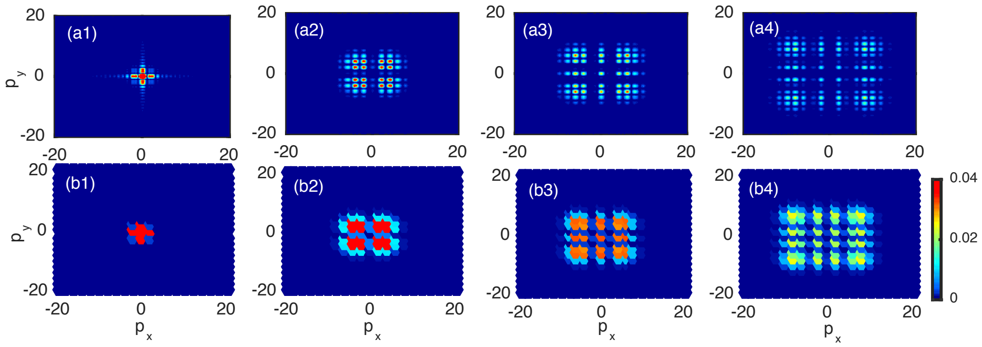

FIG. 1: (Color online) The contour plot of the atomic density distribution in (p_x, p_y) plane for in-plane lattice. (a1)-(a4) are calculated by classical treatment, and (b1)-(b4) are calculated by exact solution. Here we have $tE_{rec}/\hbar = 0.090$ and $|V/E_{rec}| = 10, 59, 88, 123$, which gives $|Vt/\hbar| = 0.900, 5.310, 7.920, 11.070$.

It is found that the density distributions calculated by these two methods are qualitatively consistent with each other, and also consistent with the experimental results [Fig. 4(a1)-(a4) in main text]. Moreover, in this case the distributions are same for red-detuning($V < 0$) and blue-detuning($V > 0$) lattice potentials.

2. Out-plane lattice with $V < 0$:

In Fig. 2 we show the density distributions for out-plane lattice with red-detuning lattice potential $V < 0$.

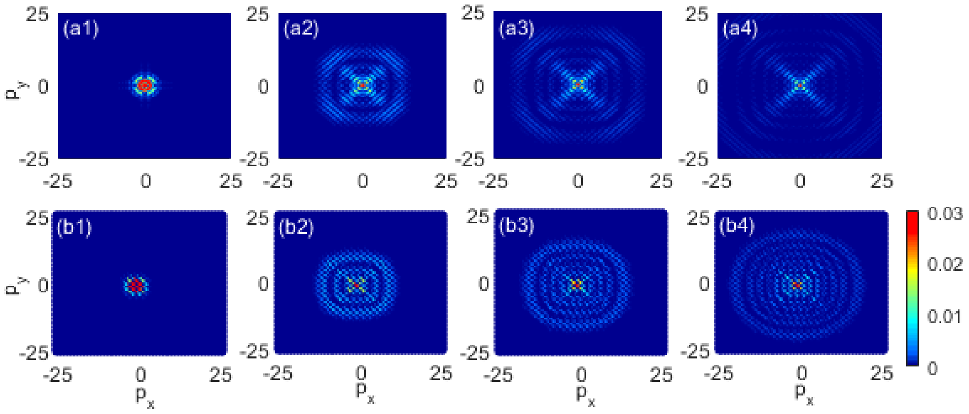

FIG. 2: (Color online) The contour plot of the atomic density distribution in (p_x, p_y) plane for out-plane lattice with $V < 0$. (a1)-(a4) are calculated by classical method, and (b1)-(b4) are calculated by exact solution. Here we have $tE_{rec}/\hbar = 0.090$ and $V/E_{rec} = -10, -59, -88, -123$, which gives $|Vt/\hbar| = 0.900, 5.310, 7.920, 11.070$.

We can see that in this case the exact results and those form classical treat are again qualitatively consistent with each other, which both show the ring structures. Meanwhile, both the size and the number of rings increase with the lattice depth. These properties are consistent with the experimental findings [Fig. 4(c1)-(c4) in main text].

3. Out-plane lattice with $V > 0$:

In Fig. 3 we show the density distributions for out-plane lattice with blue-detuning lattice potential $V > 0$.

We can see that in this case the exact results and those form classical treat show large deviations as increasing V: the classical results still give rise to bigger ring structure as V increases, identical to the $V < 0$ case in Fig. 2; while the ring structure in exact solutions gradually vanish as increasing V. Such deviation can be attributed to

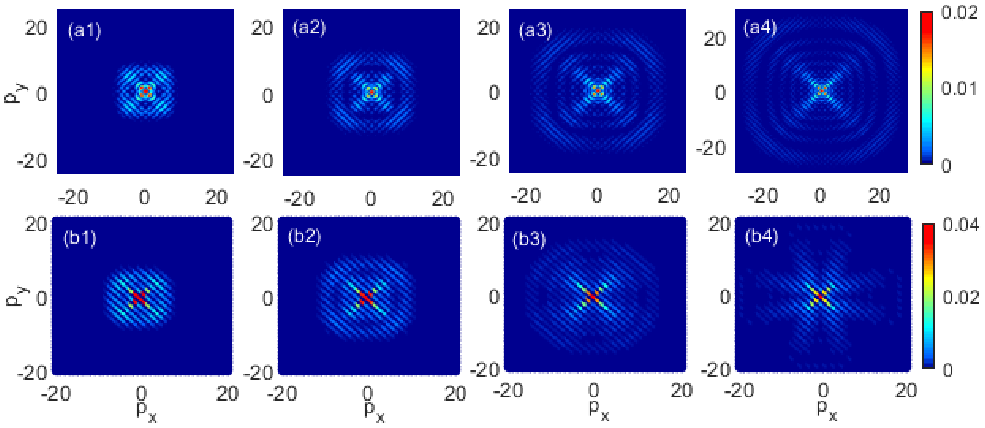

FIG. 3: (Color online) The contour plot of the atomic density distribution in (p_x, p_y) plane for out-plane lattice with $V > 0$. (a1)-(a4) are calculated by classical method, and (b1)-(b4) are calculated by exact solution. Here we have $tE_{rec}/\hbar = 0.091$ and $V/E_{rec} = 34, 53, 82, 120$, which gives $Vt/\hbar = 3.094, 4.823, 7.462, 10.920$.

the breakdown of classical treatment in describing the matter-wave dynamics under this type of potential, where the quantum fluctuations play important role in such process, as analyzed in the main text.
